# Adiponectin Deficiency Triggers Bone Loss by Up-Regulation of Osteoclastogenesis and Down-Regulation of Osteoblastogenesis

**DOI:** 10.3389/fendo.2019.00815

**Published:** 2019-11-22

**Authors:** Jihyun Yang, Ok-Jin Park, Jiseon Kim, Sora Han, Young Yang, Cheol-Heui Yun, Seung Hyun Han

**Affiliations:** ^1^Department of Oral Microbiology and Immunology, DRI, and BK21 Plus Program, School of Dentistry, Seoul National University, Seoul, South Korea; ^2^Infectious Disease Research Center, Korea Research Institute of Bioscience and Biotechnology, Daejeon, South Korea; ^3^Department of Life Science, Research Center for Cellular Heterogeneity, Sookmyung Women's University, Seoul, South Korea; ^4^Department of Agricultural Biotechnology, Research Institute for Agriculture and Life Sciences, Seoul National University, Seoul, South Korea

**Keywords:** adiponectin, osteoclast, osteoblast, adipocyte, RANKL/OPG ratio

## Abstract

Osteoporosis and bone disorders related to the metabolic syndrome are often associated with adipokines secreted by adipocytes in bone. Adiponectin, a type of adipokine, is a regulator of immune responses and metabolic processes, but its role in bone biology remains uncertain. We investigated the role of adiponectin in bone metabolism using adiponectin-deficient mice *in vivo* and *in vitro*. Adiponectin-deficient mice exhibited reduced bone mass and increased adiposity. Adiponectin-deficient calvarial cells were prone to differentiate into adipocytes rather than osteoblasts. Although bone marrow macrophages (BMMs) from adiponectin-deficient mice had low osteoclastogenic potential as osteoclast precursors with increasing interferon regulatory factor 5 expression, under co-culture conditions of calvarial cells and BMMs, the enhanced receptor activator of nuclear factor κB ligand/osteoprotegerin (RANKL/OPG) ratio of adiponectin-deficient mesenchymal progenitor cells facilitated osteoclast differentiation. In addition, increased RANKL/OPG ratio was observed in the bone marrow extracellular fluid of adiponectin-deficient mice compared to that of wild-type mice. Notably, recombinant adiponectin treatment enhanced RANKL-induced osteoclast differentiation from BMMs but up-regulated OPG production in recombinant adiponectin-exposed calvarial cells, which inhibited osteoclast differentiation. Taken together, these results suggest that adiponectin plays an inhibitory role in bone metabolism through cross talk between precursor cells of both osteoclasts and osteoblasts by regulating RANKL/OPG ratio in the bone marrow microenvironment.

## Introduction

The bone marrow microenvironment, which determines the fates of bone-resorbing osteoclasts and bone forming-osteoblasts, is an important region for bone metabolism (1). Osteoclasts, which are derived from monocyte/macrophage lineages, are regulated primarily by receptor activator of nuclear factor-κB ligand (RANKL) and osteoprotegerin (OPG), a decoy inhibitor for RANKL (2). RANKL induces and activates nuclear factor of activated T cell c1 (NFATc1) and c-Fos through MAP kinases and CREB to express osteoclastic genes such as tartrate-resistant acid phosphatase (TRAP) and cathepsin K (3). Osteoblasts, which are derived from mesenchymal stem cells, contribute to bone formation along with secretion of bone matrix by activating Runx2, osterix, or β-catenin as well as osteoclast differentiation by producing RANKL and OPG (2, 4). Under pathophysiological conditions such as osteoporosis and aging, mesenchymal stem cells are prone to differentiate into adipocytes rather than osteoblasts, resulting in increased adiposity and decreased bone formation (5).

Differentiation and activation of bone cells are regulated by soluble factors in bone marrow microenvironment. For example, bone cells and adipocytes exist in close proximity and adipokines, which are secreted mainly by adipocytes, can affect bone metabolism (6). Adipokines are differentially secreted according to the extent of adiposity and increasing adiposity is concordant with both down-regulation of adiponectin and IL-10 and up-regulation of TNF-α, IL-6, and leptin (6). IL-10 is known to promote osteoblast differentiation and inhibit osteoclast differentiation (7). Furthermore, mice lacking IL-10 develop osteopenia and decrease bone mass. In contrast, IL-6 and TNF-α suppress osteoblast differentiation and enhance osteoclast differentiation, but mice lacking these cytokines exhibit normal bone phenotypes (7). On the other hand, leptin was reported to have opposite effects on bone metabolism, which increases bone mass via induction of osteoblasts and inhibition of osteoclasts and also suppresses bone mass via the nervous system (8, 9). Thus, leptin-deficient mice exhibit either high or low bone mass, depending on skeletal region (8).

Adiponectin, a type of adipokine, is a metabolic regulator capable of increasing insulin sensitivity and fatty acid oxidation as well as an immune modulator capable of inhibiting TNF-α and IL-10 (10, 11). Adiponectin treatment ameliorates bone destruction triggered by periodontitis in both an adiponectin-deficient mouse model and a diet-induced obese mouse model (12). A number of studies using adiponectin-deficient or -overexpressing mice demonstrated controversial phenotypes (13–19). For instance, adiponectin-deficient mice exhibited a normal bone mass (14, 16), except for a slight increase of bone mass in certain age groups (16). On the other hand, adiponectin deficiency increased a bone mass (19) or induced an increased bone mass in young mice but a decreased bone mass in aged mice (17). Conversely, adiponectin deficiency showed a decreased bone mass (18). Mice overexpressing adiponectin exhibited an increased bone mass accompanied by decreased numbers of osteoclasts (13) or a reduced bone mass (15). Despite extensive research, the precise role of adiponectin in bone metabolism remains controversial and should be further investigated.

With respect to the maintenance of bone metabolism, adiponectin might be a crucial regulator in bone marrow microenvironments for the following reasons: (i) adiponectin is a plasma protein circulating in the bone marrow cavity through blood vessel (10), (ii) adiponectin is also produced by adipocytes in the bone marrow microenvironment (10), and (iii) receptors for adiponectin are expressed on both osteoclasts and osteoblasts (14). Thus, it is important to delineate the role of adiponectin in the bone marrow microenvironment. In this study, we investigated the effects of adiponectin on bone metabolism using adiponectin-deficient mice and elucidated the underlying mechanisms of adiponectin in the bone marrow environment.

## Materials and Methods

### Reagents and Antibodies

Alpha-minimal essential medium (α-MEM) and fetal bovine serum (FBS) were obtained from Gibco-BRL (Grand Island, NY, USA). Penicillin/streptomycin solution was purchased from Hyclone (Logan, UT, USA). Recombinant murine M-CSF and RANKL were purchased from Peprotech (Rocky Hill, NJ, USA). Red blood cell lysing buffer, ascorbic acid, β-glycerophosphate, dexamethasone, insulin, 3-isobutyl-1-methylxanthine (IBMX), alizarin red S (ARS), oil red O, alkaline phosphatase (ALP) kit, and TRAP staining kit were purchased from Sigma-Aldrich Chemical Co. (St. Louis, MO, USA). Anti-mouse CD16/CD32 antibody, FITC-conjugated anti-mouse CD11b antibody, PE-conjugated anti-mouse CD3ε antibody, PE-conjugated anti-mouse B220 antibody, FITC-conjugated rat IgG2b, PE-conjugated rat IgG2b, and PE-conjugated armenian hamster IgG were obtained from Biolegend (San Diego, CA, USA). Goat anti-IRF5 antibody and mouse anti-IRF5 antibody were purchased from Abcam (Cambridge, UK) for immunoblotting and for flow cytometric analysis, respectively. Cy3-conjugated anti-mouse IgG was purchased from Jackson ImmunoResearch Laboratories Inc. (West Grove, PA, USA). All other primary antibodies used for immunoblotting were obtained from Cell Signaling Technology (Beverly, MA, USA) except for antibodies against NFATc1, c-Fos (Santa Cruz Biotechnology, Santa Cruz, CA, USA), and β-actin (Sigma-Aldrich). HRP-conjugated secondary antibodies against rabbit IgG and mouse IgG were purchased from SouthernBiotech (New Orleans, LA, USA) and Sigma-Aldrich, respectively. Murine recombinant full-length adiponectin was obtained from Biovision (Mountain View, CA, USA).

### Animals and Cells

Animal experiments and care were conducted under guidelines established by the Institutional Animal Care and Use Committee of Seoul National University. All experiments were performed using 10- to 14-week-old female wild-type and adiponectin-deficient mice on the C57BL/6 background (Jackson Laboratory, Bar Harbor, ME, USA). The adiponectin-deficient Adipo^qtm1Chan^ mice donated by Dr. Lawrence Chan (Baylor College of Medicine) to Jackson Laboratory (20) were used in this study. Calvarial cells from 1-day-old C57BL/6 mouse calvaria and bone marrow-derived macrophages (BMMs) were prepared as described previously (21). To isolate peritoneal macrophages, peritoneal cells were collected from the peritoneal cavity by injecting the cavity with 5 ml of PBS and withdrawing the fluid (22). The cells were incubated for 2 h, and then the adherent cells were harvested as peritoneal macrophages. Peripheral blood mononuclear cells (PBMCs) were obtained from blood by density gradient centrifugation using Ficoll-Paque PREMIUM 1.084 (GE Healthcare Bio-Science AB, Uppsala, Sweden) according to the manufacturer's instructions. To induce osteoblast differentiation or adipocyte differentiation, calvarial cells (2 × 10^4^ cells) were plated onto 48-well plates and grown to confluence. Osteoblast differentiation was induced by incubation with 50 μM of ascorbic acid and 10 mM of β-glycerophosphate. Adipocyte differentiation was induced by incubation with 1 μM of dexamethasone, 5 μg/ml of insulin, and 0.5 mM of IBMX for the indicated time. For osteoclast differentiation, BMMs or peritoneal macrophages (3 × 10^4^ cells) were plated onto 96-well plates and incubated with 20 ng/ml of M-CSF and 40 ng/ml of RANKL for 2 to 3 days. In a co-culture experiment, calvarial cells (1 × 10^4^ cells) and BMMs (1 × 10^5^ cells) were plated onto 48-well plates and cultured in the presence of 50 nM of 1α,25-dihydroxyvitamin D_3_ (Enzo Life Science, Farmingdale, NY, USA) for 3 or 7 days.

### Cell Staining

Osteoblast differentiation was verified by staining for ALP or with ARS. For ALP staining, the cells were fixed with a fixative solution containing 26% citrate, 66% acetone, and 8% formaldehyde for 2 min, washed, and stained for ALP for 30 min. For ARS staining, the cells were fixed with 3.7% paraformaldehyde for 30 min, washed, and then stained with 2% ARS (pH 4.3) for 45 min. The ARS-stained cells were dissolved in 20% methanol and 10% acetic acid for 15 min, and then the absorbance was measured at 450 nm using a microtiter plate reader (Versa-Max, Molecular Devices, Sunnyvale, CA, USA). To analyze adipocyte differentiation, the cells were fixed with 3.7% paraformaldehyde, washed, and stained with 0.3% oil red O for 1 h. The stained cells were dissolved in isopropanol for 15 min and the absorbance was measured at 500 nm. Osteoclast differentiation was confirmed by staining for TRAP. The cells were fixed with the fixative solution, washed, and then stained for 3 min using a leukocyte acid phosphatase staining kit. TRAP^+^ MNCs with three or more nuclei were enumerated as osteoclasts.

### Micro-Computed Tomography (micro-CT) Analysis

The femora were harvested, fixed in 10% neutral buffered formalin, and scanned by X-ray micro-CT (Skyscan 1172 scanner; Skyscan, Konitch, Belgium) at 70 kV, 142 mA, 10 W, 0.5 mm aluminum filter, and 7 μm per pixel scan resolution. The micro-CT images were reconstructed by the NRecon program (Skyscan) and we created datasets in Dataviewer (Skyscan). The trabecular bone parameters were analyzed in 1 mm region in length, starting from 0.5 mm below the distal femoral growth plate using a CT-analyzer program (Skyscan). Calvaria were subjected to micro-CT at a 12 μm resolution and the bone volume was analyzed in a 3-mm diameter circle centered on an anterior fontanelle. Three dimensional (3D) images of the femora and calvaria were obtained using a CT-volume version 2.0. (Skyscan).

### Bone Histomorphometric Analysis

The femora were fixed in formalin and decalcified with 10% EDTA for 7 days. Paraffin-embedded tissue sections were prepared at the Clinical Research Institute of Seoul National University Hospital (Seoul, Korea). Sections were stained with H&E or for TRAP. Histomorphometry of the femora was analyzed with Osteomeasure software (Osteometrics, Decatur, GA, USA).

### *In vivo* Bone Destruction Analysis

GST-RANKL was prepared as described previously (23). A collagen sheet containing 5 μg of GST-RANKL was implanted on the mouse calvarial bone for 7 days. The calvarial bone was fixed in 4% paraformaldehyde and then scanned by micro-CT. Bone volume was analyzed by a CT-analyzer program (Skyscan).

### *In vitro* Bone Resorption Assay

BMMs (5 × 10^4^ cells) were plated onto calcium phosphate-coated plates (OAAS plates, Osteogenic Core Technologies, Chunan, Korea) and incubated with 20 ng/ml of M-CSF and 40 ng/ml of RANKL for 5 days. The cells were treated with 5% sodium hypochlorite for 10 min to lyse the cells, washed with distilled water, and dried. The resorbed pit area was measured using ImageJ software (version 1.44; National Institutes of Health, Bethesda, MD, USA).

### Flow Cytometric Analysis

To block non-specific binding of immunoglobulin to the Fc receptors, all cells were incubated with anti-mouse CD16/CD32 antibody for 15 min on ice before staining. To detect the expression of IRF5 in bone marrow cells, PBMCs, and peritoneal cells, the cells were stained with FITC-conjugated anti-mouse CD11b antibody for 30 min, washed, and fixed with 4% paraformaldehyde for 15 min on ice. The cells were permeabilized with 0.1% saponin for 30 min, incubated with mouse anti-IRF5 antibody for 30 min, washed, and then stained with Cy3-conjugated anti-mouse IgG for 30 min on ice. The stained cells were analyzed using FACSCalibur with CellQuest Pro software (BD Biosciences, San Diego, CA, USA). All flow cytometric data were analyzed and plotted with FlowJo software (Tree Star, San Carlos, CA, USA).

### Reverse Transcription-Polymerase Chain Reaction (RT-PCR)

The mRNA expressions of bone sialoprotein (BSP), osteocalcin (OCN), ALP, β-catenin, Runx2, PPARγ, and β-actin were determined by RT-PCR as described previously (24) with the following specific primers: BSP: 5′-GTCAACGGCACCAGCACCAA-3′, 5′-GTAGCTGTATTCGTCCTCAT-3′; OCN: 5′-AAATAGTGATACCGTAGATGCG-3′, 5′-TCTGACAAACCTTCATGTCC-3′; ALP: 5′-CCATGATCACGTCGATATCC-3′, 5′-GCCCTCTCCAAGACATATA-3′; β-catenin: 5′-GCGGCCGCGAGGTACCTGAA-3′, 5′-CAAGCCCTCGCGGTGGTGAG-3′, Runx2: 5′-CGCTCCGGCCCACAAATCTC-3′, 5′-CGCTCCGGCCCACAAATCTC-3′, PPARγ: 5′-GGGGATGTCTCACAATGCCA-3′, 5′-GATGGCCACCTCTTTGCTCT-3′; and β-actin: 5′-GTGGGGCGCCCCAGGCACCA-3′, 5′-CTCCTTAATGTCACGCACGATTTC-3′.

### Immunoblotting

BMMs (2 × 10^6^ cells) were plated onto 60 mm dishes, serum-deprived for 3 h, and then stimulated with 100 ng/ml of RANKL for 0, 5, 15, or 30 min. To detect NFATcl and c-Fos, BMMs were incubated with 20 ng/ml of M-CSF and 100 ng/ml of RANKL for 1 or 2 day(s). The cells were lysed and prepared as described previously (25). The cell lysates were separated by 10% SDS-PAGE and transferred onto PVDF membranes (Millipore, Bedford, MA, USA). After blocking with 5% skim milk in Tris-buffered saline containing 0.1% Tween-20 (TBST), the blots were probed with specific antibodies and developed using ECL Plus reagents (Neuronex Co., Pohang, Republic of Korea). The expression of IRF5 was detected in BMMs using goat anti-IRF5 antibody.

### Electrophoretic Mobility Shift Assay (EMSA)

BMMs (2 × 10^6^ cells) were plated onto 60 mm dishes and stimulated with 20 ng/ml of M-CSF and 100 ng/ml of RANKL for 1 or 2 day(s). Nuclear extracts were prepared from samples as described previously (26). Double-stranded deoxyoligonucleotide probes containing the consensus recognition sites for AP-1 and NFATc1 were as follows: AP-1: 5′-CGCTTGATGACTCAGCCGGAA-3′; NFATc1: 5′-CGCCCAAAGAGGAAAATTTGTTTCATA-3′. To confirm specific binding, each unlabeled oligonucleotide was used as a control. After electrophoresis, gels were dried and subjected to autoradiography.

### Enzyme-Linked Immunosorbent Assay (ELISA)

BMMs and peritoneal macrophages (1 × 10^5^ cells) plated onto 96-well plates were stimulated with 0.1 μg/ml of lipopolysaccharide (LPS) for 24 h and the culture supernatants were obtained. Calvarial cells were incubated alone or co-cultured with BMMs in the presence of 50 nM of 1α,25-dihydroxyvitamin D_3_ for 3 days and the culture supernatants were obtained. The bone marrow extracellular fluids were obtained by sequentially flushing tibiae with 500 μl of pre-chilled PBS and harvesting supernatant after centrifugation at 13,000 × *g* for 15 min. Serum was separated from blood by centrifugation at 13,000 × *g* for 15 min. Protein levels of IL-6, IL-10, RANKL, or OPG were determined in the culture supernatants, serum, and bone marrow extracellular fluids using a commercial ELISA kit (R&D Systems, Minneapolis, MN, USA) according to the manufacturer's instructions.

### Recombinant Adiponectin Treatment

Whole bone marrow cells (1 × 10^6^ cells) were plated onto 60 mm dishes and incubated with 2 μg/ml of adiponectin for 24 h. After removal of the remaining adiponectin, the stroma-free bone marrow cells (1 × 10^5^ cells) were plated onto 96-well plates and incubated with 20 ng/ml of M-CSF and/or 40 ng/ml of RANKL for 5 days. The calvarial cells (3 × 10^3^ cells) were plated onto 96-well plates and incubated with 2 μg/ml of adiponectin for 3 days. In a co-culture experiment, the calvarial cells (1 × 10^4^ cells) and BMMs (1 × 10^5^ cells) were plated onto 48-well plates and incubated with 2 μg/ml of adiponectin in the response of 100 ng/ml of RANKL for 7 days.

### Statistical Analysis

All experiments were performed at least two or three times and data were expressed as mean value ± standard deviation (S.D.) of three samples in each experimental group. The statistical significance of differences was evaluated by Student's two-tailed *t*-test for two independent samples and one-way ANOVA followed by a Bonferroni correction for multiple comparisons. An asterisk (^*^) indicates a significant difference in the experimental group (*P* < 0.05) compared to the control group.

## Results

### Adiponectin-Deficient Mice Exhibit Reduced Bone Mass Compared to Wild-Type Mice

**Figure 1:**
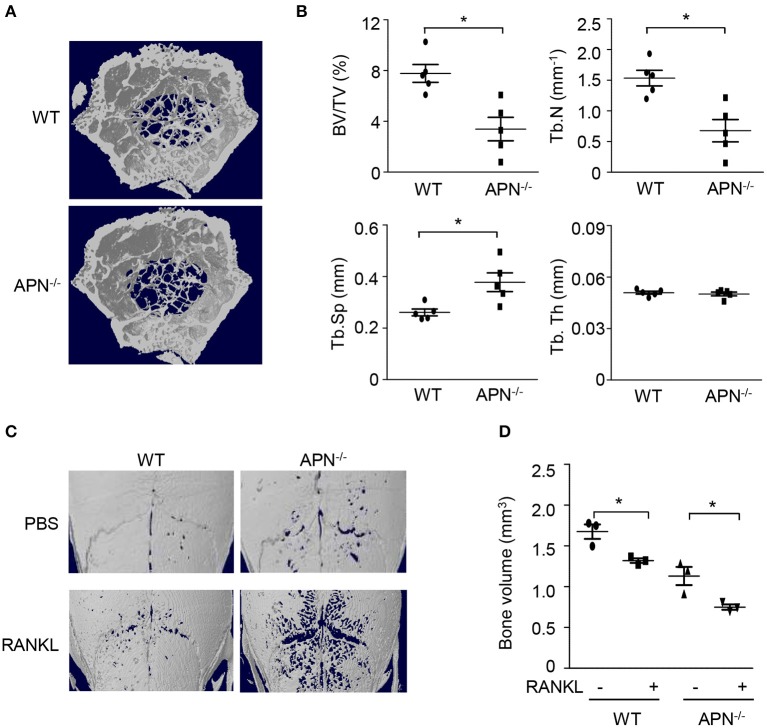
Adiponectin-deficient mice exhibit lower bone volume and higher RANKL-induced bone destruction than wild-type mice. **(A,B)** The distal femora from wild-type and adiponectin-deficient mice were scanned to obtain three dimensional micro-CT images **(A)** and trabecular bone parameters were measured using a CT-analyzer program **(B)**. BV/TV, trabecular bone volume per total bone volume; Tb.N, trabecular number; Tb.Sp, trabecular separation; and Tb.Th, trabecular thickness. **(C,D)** Collagen sheets soaked with PBS or 5 μg of GST-RANKL were implanted on mouse calvaria. At day 7, the calvaria were scanned to obtain three-dimensional micro-CT images and bone volume was analyzed by a CT-analyzer program. Data shown are the mean values ± SD of triplicate samples and are representative of three similar independent experiments.^*^P < 0.05 compared with each control. WT, Wild-type; APN^−/−^, adiponectin-deficient.

To examine whether adiponectin deficiency affects bone homeostasis, femora obtained from wild-type and adiponectin-deficient mice were analyzed using micro-CT. Trabecular bone volume and trabecular number were significantly lower and trabecular separation was higher in the femora of adiponectin-deficient mice than in those of wild-type mice (Figures 1A,B). Consistent with the results observed in the female mice, lower bone mass was observed in the adiponectin-deficient male mice than the wild-type (data not shown). When the bone volumes of calvariae were further analyzed to evaluate systemic effects of adiponectin deficiency on bone metabolism, significantly lower bone volume was also observed in the calvariae of adiponectin-deficient mice compared to those of wild-type mice in the absence or presence of RANKL (Figures 1C,D). These findings suggest that adiponectin deficiency might be a condition favoring bone destruction.

### Adiponectin-Deficient Mice Show Histomorphological Features of Reduced Bone Mass With Increased Marrow Adiposity

**Figure 2:**
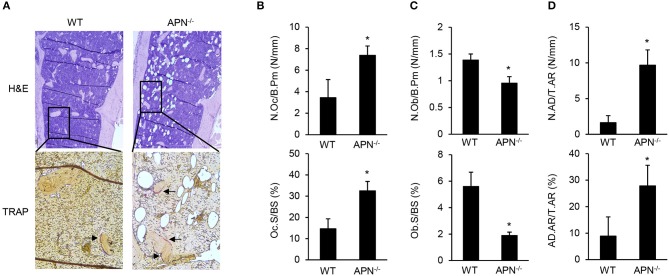
Adiponectin-deficient mice have reduced bone mass but increased adiposity. **(A)** Paraffin sections of the femur were stained with H&E or for TRAP (magnification, upper panel: ×100, lower panel: ×400). One of three similar results is shown. Histomorphometric analysis was performed for parameters related to **(B)** bone resorption, **(C)** bone formation, and **(D)** adiposity formation using Osteomeasure software. The parameters were measured with average of 15 fields of each bone (*n* = 3 per group). The arrows indicate TRAP-positive region. N.Oc/B.Pm, osteoclast number per bone perimeter; Oc.S/BS, osteoclast surface per bone surface; N.Ob/B.Pm, osteoblast number per bone perimeters; Ob.S/BS, osteoblast surface per bone surface; N.AD/T.Ar, adipocyte number per tissue areas; AD.Ar/T.Ar, adipocyte area/tissue areas. Data shown are the mean values ± SD of triplicate samples and are representative of three similar independent experiments. ^*^P < 0.05 compared with the wild-type mice.

To elucidate the precise role of adiponectin in bone metabolism, the histomorphological features of distal femora were analyzed by H&E and TRAP staining. TRAP-stained sections showed that bone resorption parameters, such as the number of osteoclasts per bone perimeter and osteoclast surface per bone surface, were significantly higher in adiponectin-deficient mice than in wild-type mice (Figures 2A,B). In contrast, bone formation parameters including the number of osteoblasts per bone perimeter and the osteoblast surface per bone surface were lower in H&E-stained sections of adiponectin-deficient mice than those of wild-type mice (Figure 2C). Notably, high marrow adiposity was observed in H&E-stained sections of adiponectin-deficient mice compared to those of wild-type mice. The number of adipocytes per tissue area or adipocyte area per tissue area were both consistently higher in adiponectin-deficient mice than wild-type mice (Figures 2A,D). These results suggest that adiponectin deficiency results in osteoporosis-like phenotypes accompanied by reduced bone mass and increased marrow adiposity.

### Adiponectin-Deficient Mesenchymal Cells Predominantly Differentiate Into Adipocytes Rather Than Osteoblasts

**Figure 3:**
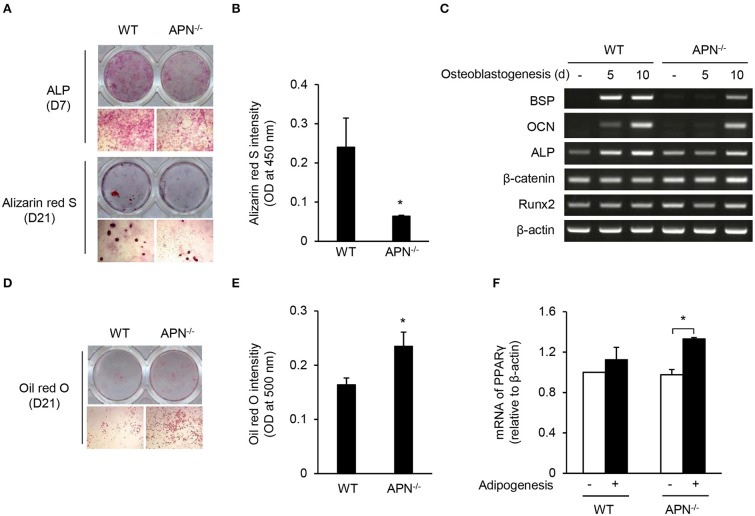
Adiponectin-deficient calvarial cells preferentially differentiate into adipocytes. **(A–C)** Mouse calvarial cells were incubated with β-glycerophosphate (10 mM) and ascorbic acid (50 μM) to induce osteoblast differentiation. **(A)** The cells were stained for ALP at day 7 and with alizarin red S at day 21 (upper, whole-well image; lower, 40× magnification). **(B)** Alizarin red S-stained cells were dissolved in 20% methanol and 10% acetic acid and the optical density (OD) was measured at 450 nm. **(C)** At days 5 and 10, total RNAs were isolated, reverse-transcribed, and subjected to RT-PCR to determine the mRNA levels of BSP, OCN, ALP, β-catenin, Runx2, and β-actin. **(D–F)** Mouse calvarial cells were incubated with dexamethasone (1 μM), insulin (5 μg/ml), and IBMX (0.5 mM) for the evaluation of adipogenic potential. **(D)** At day 21, the cells were fixed and stained using oil red O (upper, whole-well image; lower, 40× magnification). **(E)** The oil red O-stained cells were dissolved in isopropanol and the OD was measured at 500 nm. **(F)** At day 5, total RNAs were isolated, reverse-transcribed, and subjected to RT-PCR. The ratio of PPARγ to β-actin was obtained by using a densitometer. Data are mean values ± SD of triplicate samples and are representative of three similar independent experiments. ^*^P < 0.05.

Osteoblasts and adipocytes differentiate from common mesenchymal precursors (5). Because we observed low bone mass along with high marrow adipocytes in adiponectin-deficient mice, we compared the differentiation potential of mesenchymal cells obtained from wild-type and adiponectin-deficient mice using calvarial cells that have been well-established mesenchymal precursors capable of differentiating into osteoblasts as well as adipocytes (27, 28). When calvarial cells were incubated with ascorbic acid and β-glycerophosphate for osteogenesis, the degree of ALP and ARS staining appears to be lower in adiponectin-deficient cells than that of wild-type cells (Figure 3A). Quantitative analysis of ARS staining showed a substantial decrease in the mineralization during osteoblast differentiation from adiponectin-deficient cells compared to wild-type cells (Figure 3B). Concomitant with ALP and ARS analysis results, relatively low levels of BSP, OCN, and ALP mRNA expression were induced in adiponectin-deficient mesenchymal cells compared to those of wild-type cells during their osteoblastogenesis (Figure 3C). However, when calvarial cells were incubated with dexamethasone, insulin, and IBMX for adipogenesis, higher oil red O intensity was observed in adiponectin-deficient cells compared to wild-type cells (Figures 3D,E). In addition, the mRNA level of PPARγ, a major transcription factor for adipogenesis, was significantly higher in adiponectin-deficient cells than wild-type cells, though the difference was somewhat low between the two groups (Figure 3F). Taken together, these results suggest that adiponectin-deficient mesenchymal cells have potential to differentiate into adipocytes rather than osteoblasts.

### BMMs Derived From Adiponectin-Deficient Mice Exhibit Attenuated RANKL-Induced Osteoclast Differentiation and Activation

**Figure 4:**
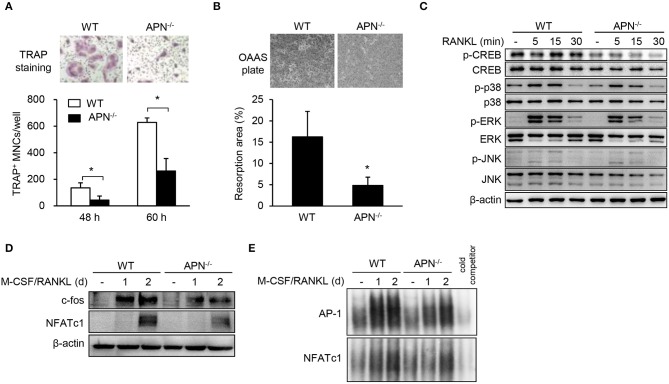
Macrophages derived from adiponectin-deficient mice exhibit attenuated RANKL-induced osteoclast differentiation. **(A)** Stroma-free bone marrow cells were incubated with M-CSF (20 ng/ml) for 5 days followed by treatment with M-CSF (20 ng/ml) and RANKL (40 ng/ml) for additional 48 or 60 h. The cells were fixed and stained for TRAP. TRAP^+^ MNCs were photographed (magnification, ×200) and enumerated. **(B)** BMMs seeded on calcium phosphate-coated plates were incubated with M-CSF (20 ng/ml) and RANKL (40 ng/ml) for 5 days. Resorbed pits were photographed (magnification, ×40) and total resorption area per well was measured with ImageJ software. **(C)** BMMs were serum-starved for 3 h and stimulated with RANKL (100 ng/ml) for 0, 5, 15, or 30 min. Then, the cells were lysed and subjected to immunoblots for the detection of phosphorylated and non-phosphorylated forms of CREB, p38, ERK, and JNK. **(D)** BMMs were incubated with M-CSF (20 ng/ml) and RANKL (100 ng/ml) for 1 or 2 days. Immunoblots were performed with antibodies specific to c-Fos and NFATc1. **(E)** BMMs were incubated with M-CSF (20 ng/ml) and RANKL (100 ng/ml) for 1 or 2 days. Nuclear extracts were incubated with ^32^P-labeled NFATc1 and AP-1 binding site-oligonucleotides. Protein-DNA binding complexes were electrophoresed and performed autoradiography. Unlabeled probe was used as a control and marked as “cold competitor.” Data are mean values ± *SD* of triplicate samples and are representative of three similar independent experiments. ^*^P < 0.05.

We hypothesized that osteoclast precursors from adiponectin-deficient mice had an effective potential to differentiate into osteoclasts based on histomorphological features (Figure 2). Thus, osteoclast precursors, BMMs, were prepared and incubated with M-CSF and RANKL for differentiation into osteoclasts. Unexpectedly, the number of TRAP^+^ MNCs was significantly lower in adiponectin-deficient cells than wild-type cells (Figure 4A) without affecting cell viability (data not shown). When BMMs were plated on calcium phosphate-coated plates and incubated with M-CSF and RANKL, the resorption area was also lower in adiponectin-deficient cells compared to wild-type cells (Figure 4B). In addition, the phosphorylation of CREB, but not MAP kinases including p38, ERK, and JNK, was scarcely induced in cells from adiponectin-deficient mice compared to those from wild-type mice upon exposure to RANKL (Figure 4C). Moreover, protein levels and DNA-binding activities of c-Fos and NFATc1 were also lower in cells from adiponectin-deficient mice than those from wild-type mice (Figures 4D,E). These results indicate that osteoclast precursors from adiponectin-deficient mice have attenuated osteoclastogenic potential.

### Attenuated RANKL-Induced Osteoclastogenic Potential Is Associated With M1-Like Properties of Adiponectin-Deficient Macrophages

**Figure 5:**
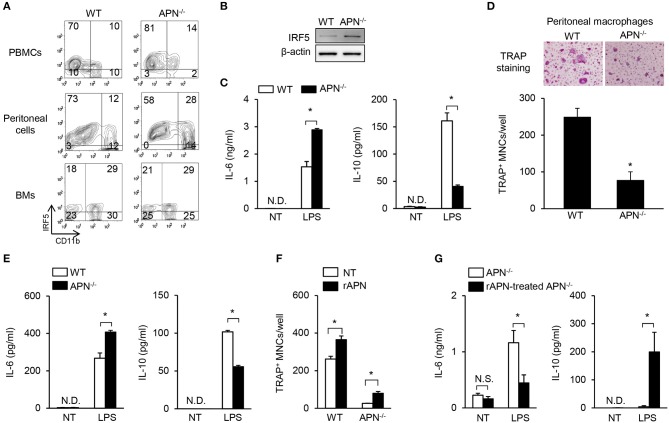
Macrophages derived from adiponectin-deficient mice have M1-like properties and attenuated osteoclastogenic potential. **(A)** PBMCs, peritoneal cells, and bone marrow cells (BMs) were blocked with anti-CD16/CD32 antibody and then stained with FITC-conjugated anti-CD11b antibody. The stained cells were fixed, permeabilized, and stained with mouse anti-IRF5 antibody followed by incubation with Cy3-conjugated anti-mouse IgG. The stained cells were analyzed using flow cytometry. Numbers indicate the percentage of each panel. One of two similar results is shown. **(B)** BMMs were differentiated from stroma free-bone marrow cells by incubation with M-CSF (20 ng/ml) for 5 days. Cell lysates were prepared and subjected to immunoblots to detect IRF5. **(C)** BMMs were stimulated with LPS (0.1 μg/ml) for 24 h. The culture supernatants were analyzed to determine the production of IL-6 and IL-10 using ELISA. **(D)** Peritoneal macrophages were isolated and incubated with M-CSF (20 ng/ml) plus RANKL (40 ng/ml) for 4 days. The cells were fixed and stained for TRAP. TRAP^+^ MNCs were photographed (magnification, ×200) and enumerated. **(E)** Peritoneal macrophages were stimulated with LPS (0.1 μg/ml) for 24 h. The culture supernatants were analyzed to determine the productions of IL-6 and IL-10 using ELISA. **(F,G)** Bone marrow cells were incubated with rAPN (2 μg/ml). After 24 h, stroma free-bone marrow cells were harvested and washed to remove the remaining rAPN. **(F)** The cells were incubated with M-CSF (20 ng/ml) and RANKL (40 ng/ml) for 3 days and stained for TRAP. The numbers of TRAP^+^ MNCs were counted. **(G)** The cells were incubated with M-CSF (20 ng/ml) for 5 days and then stimulated with LPS (0.1 μg/ml) for 24 h. The culture supernatants were analyzed to determine the production of IL-6 and IL-10. NT, non-treatment; N.D., not detected; rAPN, recombinant adiponectin; Data are mean values ± *SD* of triplicate samples and are representative of three similar independent experiments. ^*^P < 0.05.

Macrophages, which are common osteoclast precursors, can be classified into two subtypes, pro-inflammatory M1 and anti-inflammatory M2 macrophages (29). A previous report showed that peritoneal macrophages from adiponectin-deficient mice express M1 markers (30). It was recently reported that M1 macrophages have inefficient osteoclastogenic potential compared to M2 macrophages (31, 32). Thus, we examined the immunological and osteoclastogenic properties of CD11b-positive osteoclast precursors from various sources including PBMCs, peritoneal cells, and bone marrow cells. As shown in Figure 5A, the level of IRF5, a M1 specific marker, was higher in CD11b-positive populations of PBMCs and peritoneal cells obtained from adiponectin-deficient mice than those from wild-type mice, while it was similar in bone marrow cells. Additionally, the expression of IRF5 was observed in BMMs only, but not in bone marrow cells or stroma-free bone marrow cells (data now shown), indicating that fraction of IRF5-positive cells was increased by M-CSF treatment. Furthermore, compared to wild-type BMMs, adiponectin-deficient cells highly expressed IRF5 and had M1-like properties including up-regulation of IL-6 and down-regulation of IL-10 upon exposure to LPS (Figures 5B,C). When osteoclastogenic potential and macrophage phenotypes were examined using peritoneal macrophages, lower numbers of TRAP^+^ MNCs with higher IL-6 and lower IL-10 induction were also observed in adiponectin-deficient peritoneal macrophages compared to wild-type (Figures 5D,E), implying that adiponectin-deficient macrophages have attenuated osteoclastogenic potential with M1-like properties. We recently reported that M2 macrophages have higher osteoclastogenic potential than M1 macrophages (32). Since adiponectin-deficient osteoclast precursors had M1-like properties in the current study, we hypothesized that adiponectin-exposed osteoclast precursors could polarize into M2-like macrophages, thereby possessing high osteoclastogenic potential. To examine the hypothesis, bone marrow cells were pre-treated with adiponectin alone, and then the adiponectin was removed to exclude its effect in the presence of RANKL during osteoclast differentiation. Then, we determined whether adiponectin treatment affects osteoclast differentiation and macrophage polarization. As expected, when bone marrow cells were pre-treated with recombinant adiponectin and then used to induce osteoclast differentiation, the number of TRAP^+^ MNCs increased in both adiponectin-deficient and wild-type cells (Figure 5F). Moreover, upon exposure to LPS, down-regulation of IL-6 and up-regulation of IL-10 were observed in recombinant adiponectin treated cells from adiponectin-deficient mice (Figure 5G). These findings suggest that macrophages from adiponectin-deficient mice have the properties of M1 macrophages with attenuated osteoclastogenic potential.

### Adiponectin-Deficient Mesenchymal Precursors Induce Osteoclast Differentiation Through Increased RANKL/OPG Ratio

**Figure 6:**
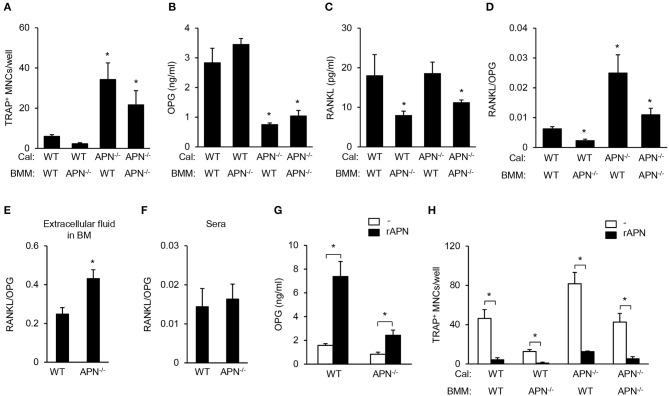
Adiponectin-deficient calvarial cells induce osteoclast differentiation through attenuated production of OPG. **(A)** Calvarial cells and BMMs were co-cultured in the presence of 1α,25-dihydroxyvitamin D_3_ (50 nM) for 7 days. After TRAP staining, the number of TRAP^+^ MNCs was measured. **(B–D)** At day 3 after co-culture, the culture supernatants were analyzed to measure the levels of **(B)** OPG and **(C)** RANKL. **(D)** RANKL/OPG ratio was calculated. ^*^P < 0.05 compared with the wild-type control. **(E,F)** Bone marrow extracellular fluids were obtained by sequentially flushing two tibiae with 500 μl of pre-chilled PBS followed by collecting the supernatant after centrifugation. Serum was separated from blood drawn from mice. The ratio of RANKL/OPG was calculated using the protein levels of RANKL and OPG in **(E)** bone marrow extracellular fluid and **(F)** serum. **(G)** Wild-type and adiponectin-deficient calvarial cells were treated with recombinant adiponectin (2 μg/ml) for 3 days and the production of OPG was measured in the culture supernatant. **(H)** Calvarial cells and BMMs were co-cultured with recombinant adiponectin (2 μg/ml) in the presence of 1α,25-dihydroxyvitamin D_3_ (50 nM) for 7 days. After TRAP staining, the number of TRAP^+^ MNCs was measured. Cal, calvarial cells. Data are mean values ± SD of triplicate samples and are representative of three similar independent experiments. ^*^P < 0.05.

Our results showed that adiponectin deficiency resulted in reduced bone mass *in vivo* but attenuated osteoclastogenic potential *in vitro*. As osteoclast differentiation can be regulated by the balance of RANKL and OPG produced by mesenchymal cells including osteoblasts (4), we examined osteoclast differentiation by co-culture of BMMs (as osteoclast precursors) and calvarial cells (as osteoblast precursors). Under co-culture conditions with adiponectin-deficient calvarial cells, all BMMs differentiated into TRAP^+^ MNCs compared to co-culture with wild-type calvarial cells (Figure 6A). OPG levels were significantly lower in BMMs co-cultured with adiponectin-deficient calvarial cells than in those with wild-type calvarial cells (Figure 6B). RANKL level was significantly lower when calvarial cells were co-cultured with adiponectin-deficient BMMs than with wild-type BMMs (Figure 6C). Importantly, the RANKL/OPG ratio, an index of osteoclast activation, was significantly higher in co-cultures with adiponectin-deficient calvarial cells than with wild-type calvarial cells (Figure 6D), implying that adiponectin-deficient calvarial cells facilitate osteoclast differentiation by increasing the RANKL/OPG ratio. Thus, we compared RANKL/OPG ratio in bone marrow extracellular fluid and sera between wild-type and adiponectin-deficient mice. Consistent with *in vitro* results, the RANKL/OPG ratio was significantly higher in bone marrow extracellular fluid obtained from adiponectin-deficient mice than from wild-type mice (Figure 6E). However, no change of the RANKL/OPG ratio in serum was observed in either group of mice (Figure 6F). In order to further examine whether adiponectin treatment affects OPG production and osteoclast differentiation, recombinant adiponectin was used to treat calvarial cells or in co-cultures of calvarial cells with BMMs. As expected, the levels of OPG increased robustly in both wild-type and adiponectin-deficient calvarial cells (Figure 6G), and the number of TRAP^+^ MNCs was significantly decreased by adiponectin treatment in co-cultures (Figure 6H). These findings indicate that adiponectin indirectly inhibits osteoclast differentiation through the induction of OPG in mesenchymal cells.

## Discussion

**Figure 7:**
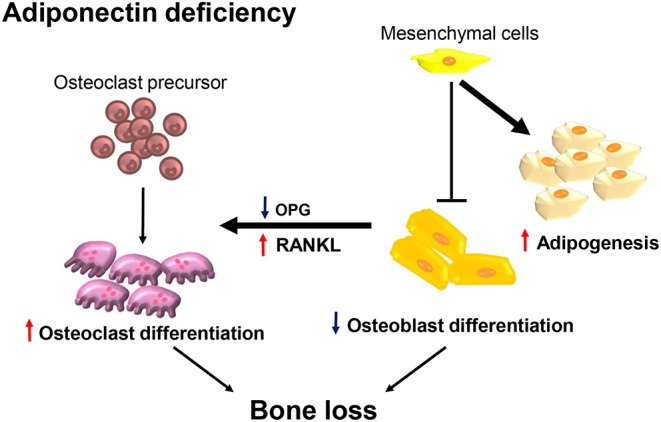
Adiponectin-deficient bone microenvironments exhibit conditions favoring bone destruction. Schematic illustration of the proposed mechanism in terms of adiponectin deficiency. Osteoclast precursors are preferentially differentiated into M1-like macrophages having attenuated osteoclast differentiation under conditions of adiponectin deficiency. However, in the adiponectin-deficient bone microenvironment, adiponectin-deficient mesenchymal lineage cells are preferentially differentiated into adipocytes rather than osteoblasts and the increased RANKL/OPG ratio is maintained. Therefore, osteoclast precursors are efficiently differentiated into active osteoclasts accompanied by increased bone destruction.

In the present study, we demonstrated that adiponectin-deficient mice exhibited osteoporosis-like phenotypes accompanied by decreased bone mass and increased marrow adiposity. Consistently, calvarial cells from adiponectin-deficient mice preferentially differentiated into adipocytes rather than osteoblasts. Although osteoclast precursors from adiponectin-deficient mice had M1-like properties and inefficient osteoclastogenic potential, adiponectin-deficient mesenchymal progenitor cells enhanced osteoclast differentiation by increasing the RANKL/OPG ratio. Potential mechanism for the bone loss at adiponectin deficiency is illustrated in Figure 7.

Macrophages from adiponectin-deficient mice had greater M1-like properties as well as lower osteoclastogenic potential when used as osteoclast precursors than did macrophages from wild-type mice. Adiponectin-deficient macrophages are seemingly inefficient for osteoclast differentiation on a per cell basis, since equal number of macrophages from wild-type and adiponectin-deficient mice was used for osteoclast differentiation assay. Our results were similar to those of previous reports showing that M1 macrophages have attenuated osteoclastogenic potential (31–33). We also showed that adiponectin treatment increased levels of M2 markers, which is in keeping with the results of a previous study (30). Importantly, the expression of IRF5, a master factor for polarizing M1 macrophages, was greater in CD11b-positive osteoclast precursors from PBMCs and peritoneal cells from adiponectin-deficient mice than in those from wild-type mice, whereas it was similar in CD11b-positive bone marrow cells. As in a previous report showing that RANKL treatment inhibits the production of M1-type cytokines (TNF-α, IL-6, and IL-12) in LPS-treated macrophages (34), we confirmed that RANKL treatment reduced IRF5 expression in adiponectin-deficient macrophages (data not shown). Therefore, we hypothesize that adiponectin inhibits the ability of osteoclast precursors to become M1 macrophages with low osteoclastogenic potential and enhances osteoclast differentiation in the bone marrow environment. A recent study reported that adiponectin treatment inhibits RANKL-induced osteoclast differentiation using BMMs derived from C57BL/6 mice (35). Despite the mice with the same genetic background, we and Wu et al. showed opposite results. We induced osteoclast differentiation from adiponectin-exposed bone marrow cells to investigate whether adiponectin affects osteoclastogenic potential of osteoclast precursors. Thus, bone marrow cells were pre-treated with adiponectin alone for 24 h and then the remaining adiponectin was removed to exclude the effect of adiponectin in the presence of RANKL during osteoclast differentiation. On the other hand, Wu et al. performed RANKL-induced osteoclast differentiation from BMMs in the presence of adiponectin, speculating that the different experimental conditions could affect the effect of adiponectin on osteoclast differentiation.

Although we found that attenuated osteoclastogenic potential was observed in macrophages from adiponectin-deficient mice, bone histomorphometric analysis of these mice showed enhancement of osteoclastogenesis. This discrepancy between osteoclastogenesis *in vivo* and *in vitro* may be explained by the increased RANKL/OPG ratio observed in co-culture systems. In cases of adiponectin deficiency, the RANKL/OPG ratio was higher in the extracellular fluid of bone marrow but not in sera, compared to the normal condition. Up-regulation of the RANKL/OPG ratio triggers excessive bone resorption leading to decreased bone mass, whereas down-regulation of RANKL/OPG attenuates bone resorption contributing to increased bone mass (36). The bone marrow microenvironment, unlike those of the serum and peritoneal cavity, is a specific niche for osteoclasts, osteoblasts, adipocytes, and their progenitors (1). Previous reports showed that adipogenic cultures of bone marrow stromal cells increased RANKL levels but decreased OPG levels (37). In addition, adipocytes were reported to directly induce osteoclast differentiation (38). That is, interactions between osteoclasts and other cells in the bone marrow environment are crucial for elucidating *in vivo* phenomena, and the results of numerous studies suggest that adiponectin is an important factor inhibiting osteoclastogenesis by down-regulating the RANKL/OPG ratio. OPG and RANKL are primarily secreted from mesenchymal cells, but RANKL production was higher in co-culture of APN^−/−^ calvarial cells with WT BMM than with APN^−/−^ BMM, suggesting that the interaction between macrophages and mesenchymal precursors could affect RANKL production.

Our results showed that adiponectin deficiency induced a low bone mass by increased RANKL/OPG ratio that is opposite of a recent report (39). Zhang et al. investigated the relationship between adiponectin and low bone mass in in patients with adolescent idiopathic scoliosis (AIS). The AIS osteopenia groups had a high level of plasma adiponectin. In particular, the cancellous bone from AIS osteopenia groups showed significantly higher levels of RANKL, RANKL/OPG ratio, and osteoclast number than the cancellous bone from normal groups. However, no significant difference in adiponectin and OPG levels was observed in the cancellous bone from control and AIS groups. Furthermore, adiponectin treatment more effectively induced RANKL expression and inhibited OPG expression in primary osteoblasts from AIS patients than in those from control groups, suggesting that high plasma adiponectin levels may affect a low bone mass through increases of RANKL/OPG ratio in osteoblasts. A previous study using human osteoblasts also reported consistent results showing that adiponectin treatment increased RANKL and suppressed OPG expression (40). In contrast, no significant difference of RANKL and OPG levels was reported in transgenic mice overexpressing human full-length adiponectin compared to wild-type mice, though the transgenic mice showed significantly higher bone mass than wild-type mice (41). Also, no significant effect of adiponectin on RANKL and OPG expression was observed in the mouse osteoblasts. Thus, the controversial effects of adiponectin on RANKL/OPG ratio might be at least partially due to differences in the experimental models and conditions.

Observed decreases in bone mass due to adiponectin deficiency are in agreement with the results of a previous report showing that adiponectin-deficient mice exhibited a low bone mineral density with high bone marrow adipocytes (18). It has been suggested that an increase of adipose tissue mass is determined by an increase of adipocyte number and size. The adipocyte number is mainly dependent on adipocyte differentiation from precursor cells, whereas the adipocyte size is associated with lipid accumulation of fully differentiated adipocytes. Our results imply that adiponectin-deficient mesenchymal precursors have more adipogenic potential, considering that the equal cell number of adiponectin-deficient and wild-type mesenchymal precursors was used for adipocyte differentiation assay. A previous report also demonstrated that adiponectin-deficient mice exhibit an increase of the number of bone marrow adipocytes compared with that of wild-type mice (18). In particular, their results showing lower bone mineral density in adiponectin-deficient mice than wild-type mice are consistent with our *in vivo* findings. However, other studies reported that adiponectin-deficient mice exhibit an increase of adipose tissue mass and adipocyte size but not adipocyte number (42) and adiponectin-overexpressing transgenic mice exhibit a decrease of adipose tissue mass and adipocyte size (43). Based on our results and compared with others, we suggest that adiponectin deficiency may facilitate not only adipocyte differentiation from mesenchymal precursors but also lipid accumulation of fully differentiated adipocytes. In the case of bone mass, some reports showed that transgenic mice overexpressing adiponectin or adiponectin-administered mice exhibited increased bone mass (13, 41), although some reports observed similar or increased bone mass in adiponectin-deficient mice compared to wild-type mice (14, 16, 19). One possible explanation for the discrepancy in results arrests upon differences in the genetic backgrounds of animals used in previous studies. In the present study we observed that adiponectin-deficient mice, considered congenic following 10 generations of backcrossing onto C57BL/6 mice, exhibited decreased bone mass but increased bone marrow adiposity, similar to the results of a previous study (44). However, such effects were not observed in adiponectin-deficient mice obtained before backcrossing (16, 17). Another possible explanation relies upon differences of physiological condition in adiponectin-deficient mice. A previous report noted that environmental factors, such as housing conditions or microflora, influence the physiological conditions of adiponectin-deficient mice (10). Bone metabolism is known to be especially affected by environmental factors such as chow, lighting, and the number of mice per cage (45, 46). Until now, clinical studies also reported controversial correlation between adiponectin level and bone loss. Some clinical studies reported that serum adiponectin is negatively associated with bone mineral density (47–50), but other studies supported a positive correlation of serum adiponectin with bone mineral density in postmenopausal women with metabolic syndrome (51) and patients with type 2 diabetes mellitus (52). Thus, further studies are needed to comprehensively address this question related to adiponectin deficiency.

The *in vivo* and *in vitro* results in this study strongly supported the hypothesis that adiponectin deficiency triggers osteoporosis-like features, accompanied by increased osteoclastogenesis, increased adipogenesis, and decreased osteogenesis. Importantly, these findings indicating that adiponectin regulates functions of bone cells call for further research to investigate reciprocal interactions among cells in the bone marrow microenvironment. Our results, taken with those of previous studies, suggest that adiponectin could function as a novel target molecule for the efficient treatment or prevention of bone destruction triggered by infection or metabolic disease.

## Data Availability Statement

All datasets generated for this study are included in the article.

## Ethics Statement

The animal study was reviewed and approved by Animal Care and Use Committee of Seoul National University.

## Author Contributions

SHH conceived the study. SHH, JY, and O-JP designed the experiments. JY, O-JP, JK, SH, and SHH performed experiments and/or interpreted data. YY and C-HY provided critical comments. All authors contributed to the discussion of the results, followed by writing and reviewing the manuscript. JY, O-JP, SHH, and C-HY contributed to the preparation of revised manuscript and figures.

### Conflict of Interest

The authors declare that the research was conducted in the absence of any commercial or financial relationships that could be construed as a potential conflict of interest.
